# Discovery of a rhamnose utilization pathway and rhamnose-inducible promoters in *Pichia pastoris*

**DOI:** 10.1038/srep27352

**Published:** 2016-06-03

**Authors:** Bo Liu, Yuwei Zhang, Xue Zhang, Chengliang Yan, Yuhong Zhang, Xinxin Xu, Wei Zhang

**Affiliations:** 1 Biotechnology Research Institute, Chinese Academy of Agricultural Sciences, Beijing 100081, China

## Abstract

The rhamnose utilization pathway in *Pichia pastoris* has not been clarified although this strain can grow well on rhamnose as a sole carbon source. In this study, four genes, *PAS_chr4_0338*, *PAS_chr4_0339*, *PAS_chr4_0340*, and *PAS_chr4_0341*, were, for the first time, predicted to be involved in rhamnose metabolism along with the previously identified gene *PAS_chr1_4-0075*. Moreover, expression of these genes, especially *PAS_chr4_0341* and *PAS_chr1_4-0075* designated as *LRA4* and *LRA3*, was confirmed to significantly increase and clearly decrease in the presences of rhamnose and glucose, respectively. *LRA4* encoding a putative L-2-keto-3-deoxyrhamnonate aldolase, was further confirmed via gene disruption and gene complementation to participate in rhamnose metabolism. Using β-galactosidase and green fluorescent protein as reporters, the promoters of *LRA4* and *LRA3* performed well in driving efficient production of heterologous proteins. By using food grade rhamnose instead of the toxic compound methanol as the inducer, the two promoters would be excellent candidates for driving the production of food-grade and therapeutically important recombinant proteins.

L-rhamnose can be utilized for growth by multitudinous microorganisms as a sole carbon and energy source^1^,^2^,^3^,^4^,^5^,^6^,^7^,^8^, and to date two different L-rhamnose metabolism pathways have been clarified in prokaryotes and eukaryotes^9^,^10^. It was further found that expression of some genes related to L-rhamnose metabolism in some microorganisms, such as *Escherichia coli*, *Listeria monocytogenes*, and *Scheffersomyces* (*Pichia*) *stipitis*, was strictly induced by L-rhamnose and strongly repressed by D-glucose^1^,^10^,^11^,^12^. Hence, an abundance of rhamnose inducible promoters were exploited and adopted to drive the expression of target genes, for example, the *E. coli* rhaP_BAD_ promoter performed excellently in the production of heterologous proteins in the presence of rhamnose^4^,^13^,^14^, and the commercial Expresso Rhamnose Cloning and Expression System was developed by the Lucigen Corporation. Therefore, rhamnose inducible promoters offer great potential for the production of target proteins and many more promoters of this kind should be exploited to satisfy the demand in basic research and for practical applications.

*Pichia pastoris*, a methylotrophic yeast, is an outstanding host for heterologous protein production. Several strong methanol-inducible promoters of genes related to the methanol utilization pathway, especially the well-characterized alcohol oxidase 1 promoter (*P*_*AOX1*_), have been frequently used to express recombinant proteins^15^,^16^,^17^. However, this promoter is not perfect for recombinant protein production due to two disadvantages of the inducer, methanol: it is highly flammable and hazardous to health. Thus, other inducible strong promoters are in high demand for controllable expression of heterologous genes in *P. pastoris*.

*P. pastoris* can grow on rhamnose as a sole carbon source, but the genes associated with L-rhamnose metabolism have not been identified. We hypothesized that the promoters of L-rhamnose metabolism-related genes would also perform well in realizing massive production of heterologous proteins. This study aimed to disclose the putative genes involved in rhamnose metabolism pathways in *P. pastoris* and to exploit the promoters of these genes for controllable production of heterologous proteins. As expected, a series of putative genes involved in L-rhamnose metabolism in *P. pastoris* was preliminarily identified and the promoters isolated from these genes, especially *PAS_chr4_0341* and *PAS_chr1-4_0075* designated as *LRA4* and *LRA3*, exhibited obvious inducibility by rhamnose. The two promoters could be exploited to enable efficient production of target proteins, especially food-grade and therapeutically important recombinant proteins, in *P*. *pastoris*.

## Results

### Putative genes involved in L-rhamnose metabolism in *P. pastoris*

**Figure 1:**
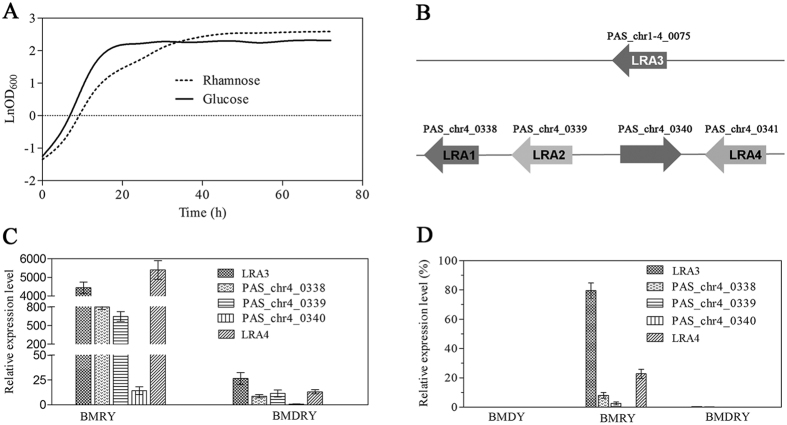
Characterization of putative genes involved in L-rhamnose metabolism in *P. pastoris*. (**A**) Growth profiles of *P. pastoris* grown on 2% (w/v) L-rhamnose or glucose as a sole carbon and energy source. (**B**) Relative positions of putative genes involved in L-rhamnose metabolism in the chromosome of *P. pastori*s. LRA1, L-rhamnose 1-dehydrogenase; LRA2, L-rhamnono-ɤ-lactonase; LRA3, L-rhamnonate dehydratase; LRA4, L-KDR aldolase. (**C**,**D**) Relative transcription profiles of these genes in *P. pastoris* grown in different media. The relative expression value of each gene in BMDY was assigned as 1 and the GAPDH gene was used as a reference (**C**); the relative expression value of the GAPDH gene was used as a reference and was assigned as 100% (**D**). Each test was conducted in triplicate and the results are presented as means ± SEM of three replicates (n = 3).

*P. pastoris* grew slightly slower on 2% (w/v) L-rhamnose than on glucose (Fig. 1A), suggesting that *P. pastoris* can utilize L-rhamnose as a sole carbon source. However, to date, genes involved in L-rhamnose metabolism have not been elucidated in *P. pastoris* except for *LRA3*, which was annotated as L-rhamnonate dehydratase after genome sequencing. By searching the nucleotide sequences of genes homologous to those of genes related to rhamnose metabolism in *S. stipitis*, the four following genes were predicted to participate in rhamnose utilization in *P. pastoris*: *PAS_chr4_0338*, *PAS_chr4_0339*, *PAS_chr4_0340*, and *LRA4*, which putatively encoded L-rhamnose 1-dehydrogenase, L-rhamnono-ɤ-lactonase, a transcription regulator, and L-2-keto-3-deoxyrhamnonate (L-KDR) aldolase, respectively (Fig. 1B).

To further investigate the relationships between theses genes and rhamnose metabolism, *P. pastoris* was cultured in BMDY, BMRY, and BMDRY media. When rhamnose was used as a sole carbon source, transcription levels of all four genes and *LRA3* increased. In particular, transcription levels of *LRA3* and *LRA4* were enhanced over 4000-fold compared to *P. pastoris* growing on glucose. When a mixture of rhamnose and glucose was used as the carbon source, the expression levels of these genes clearly decreased (Fig. 1C). The above results showed that expression of the five genes was induced by rhamnose and repressed by glucose, and also indicated that these genes are probably related to rhamnose metabolism. Moreover, transcriptional levels of *LRA3* and *LRA4* were respectively 75% and 22% of glyceraldehyde 3-phosphate dehydrogenase (*GAPDH*) transcription levels in the presence of an initial concentration of 2% rhamnose (Fig. 1D). Additionally, the above results also imply that the *LRA3* promoter (P_*LRA3*_) was strongly rhamnose-inducible, while the *LRA4* promoter (P_*LRA4*_) was weakly rhamnose-inducible.

**Figure 2:**
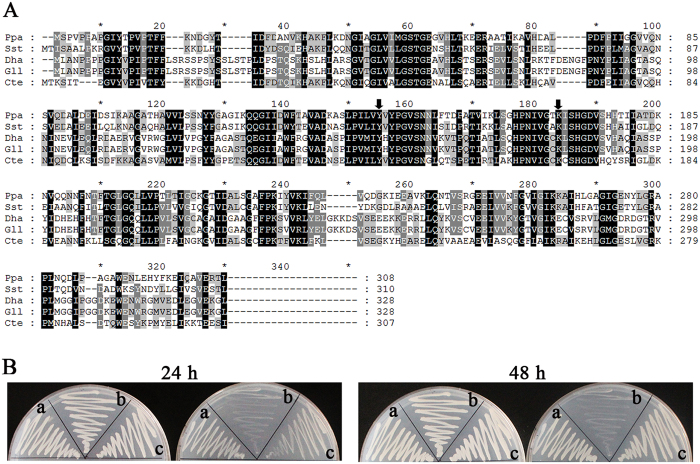
Physiological function assay of *LRA4*. (**A**) Multiple amino acid sequence alignment of L-KDR aldolases from different strains. *Ppa*, *Pichia pastoris*; *Sst*, *Scheffersomyces stipitis*; *Dha*, *Debaryomyces hansenii*; *Gll*, *Glarea lozoyensis*; *Cte*, *Candida tenuis*. The two catalytic residues (a lysine residue and a tyrosine residue) in L-KDR aldolase are indicated with arrows. (**B**) Growth profiles of the wild-type strain, *LRA4*-disrupted strain, and *LRA4*-complemented strain grown on glucose (left) or rhamnose (right) as a sole carbon source. (a) Wild-type strain, (b) *LRA4*-disrupted strain, (c) *LRA4*-complemented strain.

LRA4 is a homolog of L-KDR aldolase from *S*. *stipitis* according to amino acid sequence identity. In addition, LRA4 showed high homology when aligned with aldolases from *Debaryomyces hansenii*, *Candida tenuis* and *Glarea lozoyensis*. Furthermore, two amino acid residues (a lysine residue and a tyrosine residue) that are essential active sites in L-KDR aldolase from *S*. *stipitis*^10^ are also present in LRA4 (Fig. 2A). In order to confirm that *LRA4* was associated with L-rhamnose metabolism, a *LRA4*-disrupted strain and a *LRA4*-complemented strain were developed by gene disruption and gene complementation, respectively. The wild-type strain, *LRA4*-disrupted strain, and *LRA4*-complemented strain were grown on glucose or rhamnose as a sole carbon source. As expected, all three strains grew well on glucose, but only the wild-type strain and the *LRA4*-complemented strain grew well on rhamnose (Fig. 2B), which suggests that *LRA4* is definitely involved in L-rhamnose utilization.

### Expression of heterologous genes driven by P_*LRA3*_ and P_*LRA4*_ in *P. pastoris*

**Figure 3:**
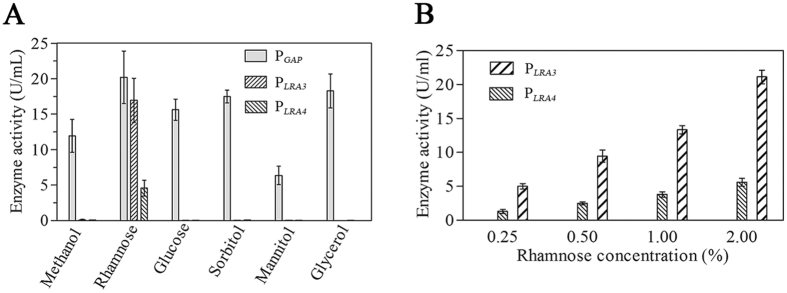
Expression profiles of β-galactosidase driven by P_*LRA3*_ and P_*LRA4*_ in the presence of different carbon sources (**A**) and various rhamnose concentrations (**B**).

Previously, *LRA4* and *LRA3* exhibited relatively high transcription activities in the presence of rhamnose. To investigate whether P_*LRA3*_ and P_*LRA4*_ were subject to strict rhamnose regulation, the effects of several carbon sources on expression of *lacB* under the control of P_*LRA3*_ and P_*LRA4*_ were monitored. Expression of *lacB* driven by P_*LRA3*_ and P_*LRA4*_ was only observed when using rhamnose as a sole carbon source. In contrast, under control of the well-characterized *GADPH* promoter (P_*GAP*_) *lacB* expression was observed when methanol, glucose, mannitol, sorbitol, and glycerol were used as sole carbon sources, in addition to rhamnose (Fig. 3A). The above results indicate that P_*LRA3*_ and P_*LRA4*_ were strict rhamnose-dependent promoters. Additionally, a dose-effect relationship occurred between rhamnose concentration (0.25–2.0%) and production of β-galactosidase driven by P_*LRA3*_ and P_*LRA4*_ (Fig. 3B), meaning that production of a target protein could be controlled in a rhamnose-dose dependent manner.

**Figure 4:**
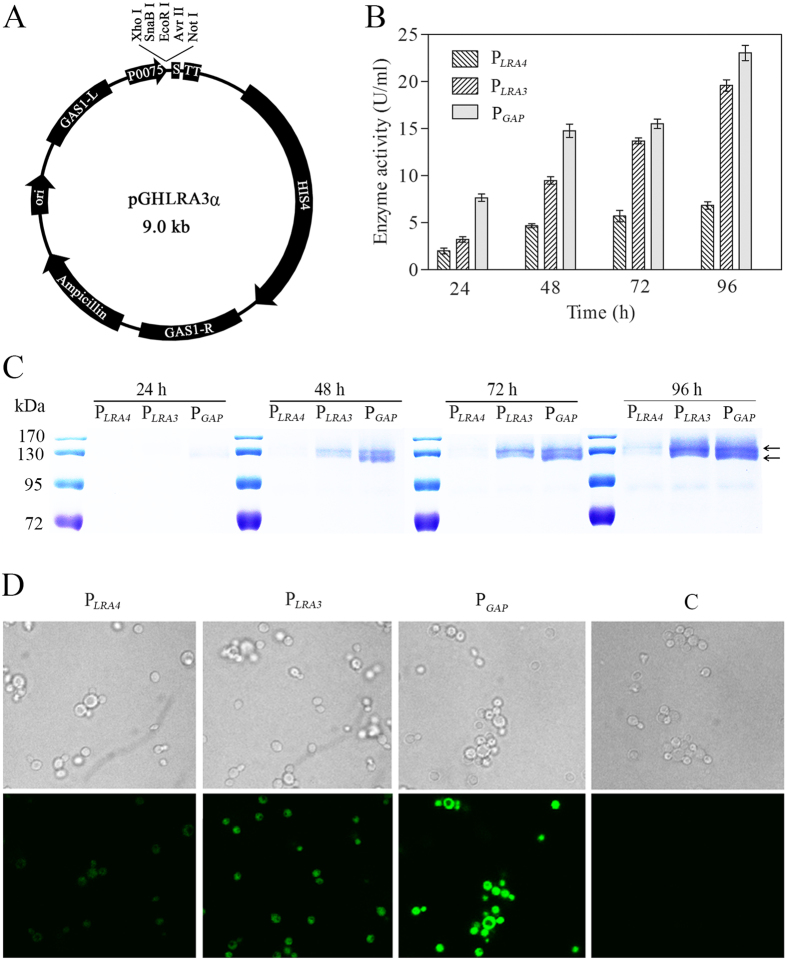
Expression profiles of heterologous genes driven by P_*LRA3*_ and P_*LRA4*_ in *P. pastoris*. (**A**) Schematic representation of plasmid pGHLRA3α for heterogeneous gene expression in *P. pastoris*. (**B**) β-galactosidase activity under the control of P_*LRA3*_, P_*LRA4*_, and P_*GAP*_ at various time points. (**C**) Production of recombinant β-galactosidase using P_*LRA3*_, P_*LRA4*_, and P_*GAP*_ for various durations. (**D**) Green fluorescence under the control of P_*LRA3*_, P_*LRA4*_, and P_*GAP*_; ‘C’ represents *P. pastoris* GS115, which was used as a control.

To validate the efficiency of the rhamnose-inducible P_*LRA3*_ and P_*LRA4*_ in target gene expression, P_*GAP*_ and the two identified rhamnose-inducible promoters were subjected to a comparative protein production study using β-galactosidase and green fluorescent protein (GFP) as reporters. Vectors pGHLRA3α, pGHLRA4α, and pGHGAPα based on P_*LRA3*_, P_*LRA4*_, and P_*GAP*_, respectively, were developed, with pGHLRA3α illustrated as an example in Fig. 4A. One reporter gene, *lacB*, encoding a β-galactosidase, was ligated into the above vectors via the *Sna*BI and *Not*I restriction sites to generate pGHLRA3αLacB, pGHLRA4αLacB, and pGHGAPαLacB, in which *lacB* expression was controlled by P_*LRA3*_, P_*LRA4*_, and P_*GAP*_, respectively, and was then integrated into the *gas1* locus of the *P. pastoris* chromosome after transformation. The β-galactosidase activities in culture supernatants of the three recombinant *P. pastoris* strains grown in BMRY and BMDY were monitored. It was observed that β-galactosidase activities in supernatants of the different cultures in BMRY increased with incubation duration and reached a maximum at 72 h: 23 U/ml, 18 U/ml, and 6 U/ml for recombinant strains harboring pGHLRA3αLacB, pGHLRA4αLacB, and pGHGAPαLacB, respectively (Fig. 4B). The target protein productions under the control of P_*LRA3*_ and P_*LRA4*_ in the presence of rhamnose were approximately 80% and 26% of that under P_*GAP*_, respectively, which was consistent with the transcription levels determined by real-time PCR. Production of β-galactosidase in culture supernatants of the different recombinant strains was assayed via sodium dodecyl sulfate polyacrylamide gel electrophoresis (SDS-PAGE) (Fig. 4C).

To investigate whether the two promoters also drove efficient expression of other genes, expression of another reporter gene, GFP (*gfp*), was also monitored. For intracellular expression of *gfp*, three new vectors, pGHLRA3, pGHLRA4, and pGHGAP, were developed without a protein secretion signal based on vectors pGHLRA3α, pGHLRA4α, and pGHGAPα. *gfp* was ligated into vectors pGHLRA3, pGHLRA4, and pGHGAP via the *EcoR*I and *Not*I restriction sites, and was integrated into the *gas1* locus of the *P. pastoris* chromosome. Green fluorescence was monitored after the recombinant strains were grown in BMRY. Fluorescence was observed in all three recombinant strains, in which *gfp* expression was regulated by P_*GAP*_, P_*LRA3*_, and P_*LRA4*_. Mean green fluorescence intensity was 235 (P_*LRA4*_), 563 (P_*LRA3*_) and 2249 (P_*GAP*_) respectively, which indicated that the strengths of the three promoters were ranked as follows: P_*GAP*_ (high), P_*LRA3*_ (middle), and P_*LRA4*_ (weak) (Fig. 4D). According to the above results, P_*LRA3*_ would be more advantageous for inducible expression of target genes than P_*LRA4*_.

### Characterization of putative P_*LRA3*_

**Figure 5:**
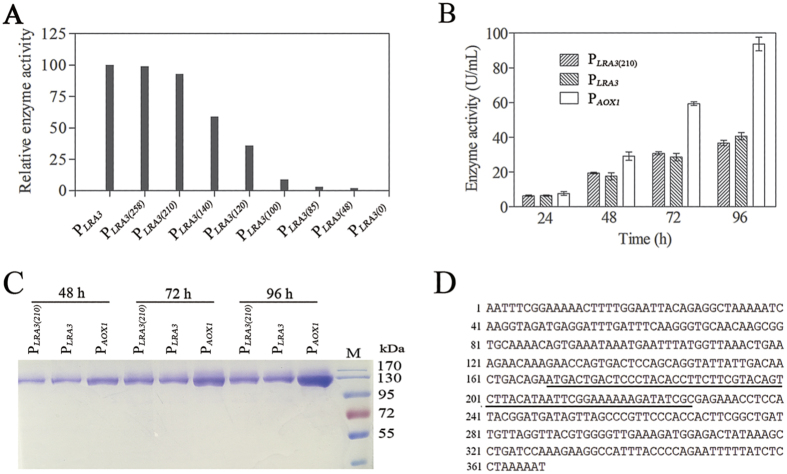
Identification of the *LRA3* minimal promoter. (**A**) Promoter activities of all 5′-deletion promoter constructs were evaluated according to β-galactosidase activity in culture supernatant of recombinant strains grown in BMRY. Numbers refer to the number of nucleotides remaining in the 5′-flanking region upstream of the ATG of the initiating methionine of *LRA3*. (**B**) β-galactosidase activity and (**C**) β-galactosidase production in culture supernatant of recombinant strains harboring the indicated promoter to drive *lacB* expression at different induction time. (**D**) The nucleotide sequence of putative P_*LRA3*_ and the region between −140 to −210 bp (underlined). Each test was conducted in triplicate and the results are presented as means.

P_*LRA3*_, a newly discovered strong rhamnose-inducible promoter, could potentially be widely used to drive production of proteins of interest in *P. pastoris*. To identify the minimal promoter region, 5′-deletion constructs of P_*LRA3*_ were derived from pGHLRA3αLacB and were inserted into pGHLRA3αLacB between the *Asc*I and *SnaB*I sites, generating pGHLRA3(258)αLacB, pGHLRA3(210)αLacB, pGHLRA3(140)αLacB, pGHLRA3(120)αLacB, pGHLRA3(100)αLacB, pGHLRA3(85)αLacB, pGHLRA3(48)αLacB, and pGHLRA3(0)αLacB. These deletion constructs were transformed into *P. pastoris* and the β-galactosidase activities in culture supernatants of the different recombinant strains were determined. The results showed that a promoter harboring a 5′ flanking region greater than 210 bp upstream of the ATG of the initiating methionine of *LRA3* was sufficient for transcriptional activation (Fig. 5A), which suggests that a 210-bp DNA fragment harbors all the necessary elements to express recombinant proteins in *P. pastoris*. The promoter with a 5′ flanking region containing the 140 bp upstream of the translation start site was not as efficient, indicating that elements in the DNA region between −140 bp and −210 bp were essential to efficient expression of target genes. Additionally, some important regulatory elements regulating gene expression occur between −100 bp and −140 bp, as the deletion of this region resulted in an obvious decrease in β-galactosidase activity (Fig. 5A).

The efficiency of P_*LRA3(210)*_ and P_*LRA3*_ was further determined using β-galactosidase as a reporter gene and P_*AOX1*_ as a positive control promoter. The β-galactosidase activities in culture supernatants of recombinant strains harboring pGHLRA3αLacB, pGHLRA3(210)αLacB and pPIC9LacB increased during the induction. At 96 h, activity and production of β-galactosidase in culture supernatants of strain harboring pGHLRA3αLacB and pGHLRA3(210)αLacB were almost same, and were less than half of that of strain harboring pPIC9LacB (Fig. 5B,C). The results further indicated that P_*LRA3*_ was a middle strong rhamnose-inducible promoter. The nucleotide sequence of P_*LRA3*_ was shown and the region between −140 to −210 bp was underlined (Fig. 5D).

## Discussion

Identification of genes related to rhamnose metabolism in *P. pastoris* allowed us to further elucidate the rhamnose metabolism pathway and to exploit inducible promoters for controllable expression of target genes in *P. pastoris*. In this study, a set of genes involved in rhamnose metabolism in *P. pastoris* was first predicted according to the nucleotide sequence identity to genes in *S. stipitis*. Then, *LRA4* was further confirmed to correlate with rhamnose metabolism based on the result that *LRA4* -disrupted *P*. *pastoris* did not grow on rhamnose as a sole carbon source, while the wild-type strain and the *LRA4*-complemented strain did. To further confirm the physiological role of this gene, the biochemical characterization of the enzyme encoded by *LRA4* was examined in another study. Similarly, the physiological roles of the other genes we identified should also be verified via gene disruption and complementation and biochemical function assays in future studies. Additionally, it was noted that the genes encoding the enzymes for rhamnose metabolism in *P. pastoris* did not cluster. Three genes (*PAS_chr4_0338*, *PAS_chr4_0339*, and *LRA4*) and the regulator gene (*PAS_chr4_0340*) were organized in a gene cluster, while *LRA3* was independent. This is not the same organization found in *S. stipitis*, in which a set of genes correlated with rhamnose catabolism clustered in a rhamnose operon. The difference reflects different evolutionary processes in rhamnose metabolism between *S. stipitis* and *P. pastoris*.

P_*AOX1*_, which is strictly and strongly activated by methanol, was the promoter most widely used for production of heterologous proteins in *P. pastoris*. However, strong promoters that produce heterologous proteins in high quantities are unsuitable for certain situations, such as production of active products that are highly dependent on correct folding or processing in the secretory pathway^18^ or the expression of various lethal or toxic genes; in these cases weaker promoters have been reported to be more feasible^19^,^20^. Also, in addition to using the toxic compound methanol as the inducer, P_*AOX1*_-based fermentation includes the following disadvantages: 1) relatively long fermentation duration and 2) sophisticated feeding strategies including but not limited to, *i*) initial culture of recombinants in buffered glycerol medium, followed by *ii*) harvesting and resuspending cells in methanol medium. To broaden the applications of *P. pastoris* and simplify the process of fermentation, alternative promoters that do not require methanol are in demand, and various promoters have been described^21^. Five rhamnose-inducible genes were discovered in our study, and among them P_*LRA3*_ and P_*LRA4*_ were strictly activated in the presence of rhamnose and almost completely repressed by glucose. P_*LRA3*_ in the presence of rhamnose was confirmed to be comparable to the typical strong promoter, P_*GAP*_, which has been commonly used to drive efficient expression of target genes in *P. pastoris*. Due to using rhamnose instead of the flammable and toxic compound methanol as an inducer, P_*LRA3*_ could be used for substantial production of recombinant proteins in the food industry and production of therapeutically important recombinant proteins for commercial applications. As for P_*LRA4*_, it was much weaker than P_*LRA3*_ and is a better candidate for certain academic purposes, for example, controllably expressing potentially lethal or toxic genes that affect cell viability. Few promoters isolated from genes related to sugar metabolism have been reported in *P. pastoris* and, to our knowledge, the sorbitol dehydrogenase promoter, a constitutively expressed promoter with a transcription strength similar to P_*GAP*_, is the only one that had been intensively investigated prior to our study. Compared to the sorbitol dehydrogenase promoter, P_*LRA3*_ is disadvantageous in its transcription activity, however P_*LRA3*_ is advantageous for controllable expression of heterologous genes, including some genes in which the products might be toxic to the host cell.

## Materials and Methods

### Bacterial strains and media

*E. coli* Trans1-T1 (TransGen, Beijing, China) and *P. pastoris* GS115 (Invitrogen, Carlsbad, CA, USA) were used as the hosts for gene cloning and expression, respectively. The *pEasy*-Blunt Simple Cloning Vector was purchased from TransGen.

MD medium contained 1.34% yeast nitrogen base (YNB), 4 × 10^−5^% biotin, 2% dextrose, and 2% agar; BMDY, BMRY, and BMDRY media (pH 6.0) contained 300 mM potassium phosphate, 1.34% YNB, 4 × 10^−5^% biotin, and 4 × 10^−3^% histidine, plus either 2% dextrose (BMDY), 2% rhamnose (BMRY), or 1% dextrose and 1% rhamnose (BMDRY).

The primers used for PCR are listed in Supplementary Table S1.

### Total RNA preparation and real-time PCR

*P. pastoris* GS115 was grown in BMDY, BMRY, or BMDRY to OD_600_ 1.2–1.4, and then cells were harvested by centrifugation (12,000 g × 5 min) at 4 °C and immediately stored at −80 °C. Total RNA from *P. pastoris* cells was extracted with Trizol reagent (Invitrogen) followed by digestion with DNase I (RNase-free; MBI Fermentas, St. Leon-Roth, Germany) to remove trace DNA. cDNA was synthesized from each RNA sample (1 μg) using the RevertAid H Minus First Strand cDNA Synthesis Kit (K1631; MBI Fermentas). Real-time PCR assays were performed in the IQ5 Real-Time PCR Detection System (Bio-Rad Laboratories, Hercules, CA) using the SuperReal PreMix SYBR Green I Kit (Toyobo, Osaka, Japan). Primers used for real-time PCR are listed in Table S1. The GAPDH gene was used as a reference. All samples were analyzed in triplicate and the data are presented as means ± standard deviation (SD).

### Construction of a homologous recombination vector in *P. pastoris*

The genomic DNA was isolated from *P. pastoris* cells using the TIANamp Yeast DNA Kit (Tiangen, Beijing, China). Based on the genome sequence of *P. pastoris* GS115, DNA fragments upstream and downstream of the open reading frame of *gas1* were amplified from genomic DNA by two pairs of primers (*gas1*-F1 and *gas1*-R1, *gas1*-F2 and *gas1*-R2, respectively). HIS4, a selectable marker for isolating *Pichia* recombinant strains, was amplified from plasmid pPIC9 using the primers *his4*-F and *his4*-R. The three abovementioned DNA fragments were ligated into a DNA fragment named “GH” via overlap-extension PCR with the primers *gas1*-LF and *gas1*-RR. In addition, a DNA fragment designated “OA” containing the origin of replication derived from pBR322 and an ampicillin resistance gene was amplified from plasmid pPIC9 with primers OA-F and OA-R. Finally, the two DNA fragments, GH and OA, were ligated into a circular molecule by homologous recombination using the CloneEZ Kit (GenScript, Nanjing, China), generating the recombination vector pGH01. pGH01 was integrated into the *gas1* locus of the *P. pastoris* chromosome via homologous recombination.

### *LRA4* disruption and complementation

DNA fragments upstream and downstream of *LRA4*, which were used as homologous arms for integration of target genes into the *LRA4* locus, were amplified from genomic DNA of *P. pastoris* GS115. The expression cassette of a zeocin resistance gene was obtained from the plasmid pPICzα. The three PCR fragments were fused by overlap-extension PCR to generate a gene disruption cassette for disruption of *LRA4* in *P. pastoris*. From the resultant DNA fragments, 500 ng were transformed into *P. pastoris* GS115 via electroporation. Transformants were first screened on yeast extract-peptone-dextrose (YPD) agar plates containing 100 μg/ml zeocin and then verified by PCR.

To complement *LRA4*, the complementation plasmid pGH/*LRA4* was developed as follows. A DNA fragment harboring full length *LRA4* was cloned from genomic DNA of *P. pastoris* GS115 and then ligated into the pGH01 vector via the *Asc*I and *Pac*I restriction sites to generate the pGH/*LRA4* plasmid. pGH/*LRA4* was introduced into *P. pastoris* GS115 with *LRA4*-disruption via electroporation after linearization by the restriction enzyme *Swa*I. Transformants were screened on MD medium and then verified by PCR and DNA sequencing.

### Expression of exogenous genes under P_*LRA3*_ and P_*LRA4*_ in *P. pastoris*

DNA fragments containing the putative promoter regions of *LRA3*, *LRA4*, and *GAPDH* were obtained from the genomic DNA of *P. pastoris*. The DNA fragment comprising the protein secretion signal, multiple cloning site, and transcription termination was cloned from plasmid pPIC9. The two DNA fragments were fused by overlap-PCR to generate the exogenous gene expression cassette in which exogenous gene expression was driven by P_*LRA3*_, P_*LRA4*_ and P_*GAP*_, and then the cassettes were respectively ligated into vector pGH01 via the *Asc*I and *Pac*I restriction sites to generate expression vectors pGHLRA3α, pGHLRA4α, and pGHGAPα. Additionally, three vectors, pGHLRA3, pGHLRA4, and pGHGAP, were developed without the protein secretion signal in the same manner as pGHLRA3α, pGHLRA4α, and pGHGAPα.

The *lacB* gene (GenBank accession number: AJ431643.1), which was cloned from the *Aspergillus candidus* chromosome and encodes a β-galactosidase, was ligated into pGHLRA3α, pGHLRA4α, and pGHGAPα and pPIC9 via the *Sna*BI and *Not*I restriction sites. The *gfp* gene (GenBank accession number: JQ733033.1) encoding green fluorescent protein was synthesized by GenScript Biotechnology Co. Ltd. and was ligated into pGHLRA3, pGHLRA4, and pGHGAP via the *EcoR*I and *Not*I restriction sites.

### Generation of 5′-serially deleted P_*LRA3*_

The 5′-serially deleted P_*LRA3*_ constructs were generated by PCR using pGHLRA3αLacB as a template. The DNA fragment harboring P_*LRA3*_ and partial *lacB* in pGHLRA3αLacB was replaced by the 5′-serially deleted promoters and partial *lacB* between restriction sites *Asc*I and *Sna*BI to generate plasmids pGHLRA3(256)αLacB, pGHLRA3(210)αLacB, pGHLRA3(140)αLacB, pGHLRA3(120)αLacB, pGHLRA3(100)αLacB, pGHLRA3(85)αLacB, pGHLRA3(48)αLacB, and pGHLRA3(0)αLacB.

### *lacB* expression and enzyme assays

To evaluate the strength of P_*LRA3*_, P_*LRA4*_ and P_*GAP*_, a single colony of different recombinant *Pichia* cells was inoculated into in YPD medium and gown overnight at 28 °C with vigorous shaking (200 rpm). The cultures were inoculated into fresh BMRY medium at 1% (v/v) and grown at 28 °C with vigorous shaking (200 rpm).

To evaluate the strength of P_*LRA3*_, P_*LRA3(210)*_ and P_*AOX1*_, a single colony of different recombinant *Pichia* cells was inoculated into in 50-ml YPD medium at 28 °C for 48 h in shaking flasks. The cells with *lacB* expression under the control of P_*LRA3*_ and P_*LRA3(210)*_were harvested and then grown in 50-ml BMRY medium, and the cells with *lacB* expression under the control of P_*AOX1*_ were harvested and then grown in 50-ml BMMY medium with addition of methanol to a final concentration of 1.0% [v/v] at a interval of 24 h. β-galactosidase activity and production in culture supernatant were monitored at intervals.

The β-galactosidase activity was assayed as described by Nie *et al.*^15^, with minor modification. The reaction system containing 200 μl of enzyme solution and 800 μl of 0.25% (w/v) ortho-nitrophenyl-β-galactoside (*o*NPG) in pre-incubated (60 °C for 5 min) phosphate-citrate buffer (50 mM, pH 5.2) was incubated at 60 °C for 15 min, followed by sequential addition of 1 ml of 10% trichloroacetic acid and 2 ml of 1 M Na_2_CO_3_. The absorbance at 420 nm was measured. One unit of β-galactosidase was defined as the amount of enzyme that released 1 μmol of *o*-nitrophenol per minute under standard conditions (pH5.2, 60 °C, 15 min).

### *gfp* expression and assays of green fluorescence intensity

A single colony of recombinant *Pichia* cells was inoculated into in YPD medium and gown overnight at 28 °C with vigorous shaking (200 rpm). The cultures were inoculated into fresh BMRY medium at 1% (v/v) and grown for 48 h at 28 °C with vigorous shaking (200 rpm). Recombinant *Pichia* cells were then collected, washed three times, and resuspended in 0.9% NaCl. Green fluorescence intensity in recombinant cells was determined using a laser scanning confocal microscope (Nikon, Japan) with 2s per image to collect a 1024 × 1024 pixel image.

## Additional Information

**How to cite this article**: Liu, B. *et al.* Discovery of a rhamnose utilization pathway and rhamnose-inducible promoters in *Pichia pastoris*. *Sci. Rep.* **6**, 27352; doi: 10.1038/srep27352 (2016).

## Supplementary Material

Supplementary Information
