# Supplementary material for: Discovery of a rhamnose utilization pathway and rhamnose-inducible promoters in Pichia pastoris
Source: Sci Rep. 2016 Jun 3;6:27352. doi: 10.1038/srep27352 (PMC4891683; doi:10.1038/srep27352)
Supplement: Supplementary Information [file srep27352-s1.pdf]

Discovery of a rhamnose utilization pathway and rhamnose-inducible promoters in *Pichia pastoris*

Bo Liu[#], Yuwei Zhang[#], Xue Zhang, Chengliang Yan, Yuhong Zhang, Xinxin Xu, Wei Zhang*

Biotechnology Research Institute, Chinese Academy of Agricultural Sciences, Beijing 100081, China

*Corresponding author: Biotechnology Research Institute, Chinese Academy of Agricultural Sciences, No. 12 Zhongguancun South Street, Beijing 100081, China.

Tel./Fax: +86 10 82106141, E-mail: zhangwei02@caas.cn

[#] These authors contributed equally to this work.

Table S1 Primers used in this study

Primer	Sequences (5’ to 3’)	Primers used for
gas1-F1	CATTTCTGCTGTCATAACCGAGATAGCACAACAATAAACCGCTTCG	Amplifying left nucleotide sequence of *gas1* locus
gas1-R1	GTGTCAGTAGAGACTAGAGCATGGCGCGCCCAAGCACAGCAGTGTAACCGTC	Amplifying left nucleotide sequence of *gas1* locus
his4-F	GCCATGCTCTAGTCTCTACTGACACTGATCATTAATTAAGTCCATTCCGACAGCATCG	Amplifying a selectable marker for isolating *Pichia* recombinant strains from plasmid pPIC9
his4-R	ATCATAGATACTCGACGCTCTCCC	Amplifying a selectable marker for isolating *Pichia* recombinant strains from plasmid pPIC9
gas1-F2	GGGAGAGCGTCGAGTATCTATGATTGAAGTTATCACCAACAAGTCCAAT	Amplifying right nucleotide sequence of *gas1* locus
gas1-R2	GCTGACTCATGTTGGTATTGTGAAACTGGCAAGCAGCCTGTTCT	Amplifying right nucleotide sequence of *gas1* locus
OA-F	TTCACAATACCAACATGAGTCAGC	Amplifying the origin of replication derived from pBR322

		and ampicillin resistance gene from plasmid pPIC9
OA-R	TATCTCGGTTATGACAGCAGAAATG	Amplifying origin of replication derived from pBR322 and ampicillin resistance gene from plasmid pPIC9
LRA4-KO-F1	GGTTTTGGAATCCAACGATCC	Amplifying left homologous arm of *LRA4* locus
LRA4-KO-R1	TTTTGAAGCTATGGTGTGTGGGGCTGGTATCAAGCAACAAGGTATC	Amplifying left homologous arm of *LRA4* locus
LRA4-KO-F2	CGAAGGCTTTAATTTGCAAGCTTCGATCTCATCCAGGGCATC	Amplifying right homologous arm of *LRA4* locus
LRA4-KO-R2	CAGCATCCAGCCCTTTTGAC	Amplifying right homologous arm of *LRA4* locus
zeoncin-F	CCCACACACCATAGCTTCAA AA	Amplifying zeocin resistant gene from plasmid pPICzα
zeoncin-R	GCTGGTATCAAGCAACAAGGT	Amplifying zeocin resistant gene from plasmid pPICzα
LRA4-V-F	TTATTACATCCAACGGTGATACTCTC	Verifying the discruption of *LRA4*
LRA4-V-R	GAATGCACGGTAAAGAATGTCAC	Verifying the discruption of *LRA4*
LRA4-C-F	GCATCGGCGCGCCTTTGAGAGATTTTAATTCAACGAACTAC	Amplifying a DNA fragment containing *LRA4*
LRA4-C-R	GATCATTAATTAAGCTTTTATTACATCCAACGGTGA	Amplifying a DNA fragment containing *LRA4*

LRA3-F	TTCAGTGTCATCTGGGTGCAAC	Real-time PCR for *LRA3*
LRA3-R	CCTGACTTCCCACTGATGGTAGAC	Real-time PCR for *LRA3*
0338-F	CACTGATACTGGACACTCCGATG	Real-time PCR for *PAS_chr4_0338*
0338-R	GAGATGCCTGCTGAAACTTACTTG	Real-time PCR for *PAS_chr4_0338*
0339-F	TTCAATCCACCGAGCCACAG	Real-time PCR for *PAS_chr4_0339*
0339-R	CCAGCTCTTTCTGCCCGTATC	Real-time PCR for *PAS_chr4_0339*
0340-F	GAGTTTCTCAGTCTCACAGCCTTG	Real-time PCR for *PAS_chr4_0340*
0340-R	CGTATAGCACGGACCTAGCACTC	Real-time PCR for *PAS_chr4_0340*
LRA4-F	CTTGAATCTCCTTGAAGTAGTGCTC	Real-time PCR for *LRA4*
LRA4-R	GGCGTCATTGGAATCAAGAAG	Real-time PCR for *LRA4*
P_{LRA3}-F	GTAACGGCGCGCCAATTTCGGAAAAACTTTTGGAATTA	Amplifying a DNA fragment containing *LRA3* promoter

P_{LRA3}-R	GCAGTAAAAATTGAAGGAAATCTCATATTTTTAGGAGATAAAAATTC TGGGG	Amplifying a DNA fragment containing *LRA3* promoter
P_{LRA4}-F	GTAACGGCGCGCCTTTGAGAGATTTTAATTCAACGAACTAC	Amplifying a DNA fragment containing *LRA4* promoter
P_{LRA4}-R	GCAGTAAAAATTGAAGGAAATCTCATTGGTTGGAGTAACCGGGAAG	Amplifying a DNA fragment containing *LRA4* promoter
P_{GAP}-F	GTAACGGCGCGCCTCTTTTTTGTAGAAATGTCTTGGTGTC	Amplifying a DNA fragment containing *GAP* promoter
P_{GAP}-R	GCAGTAAAAATTGAAGGAAATCTCATCGTTTCGAAATAGTTGTTCAA TTGA	Amplifying a DNA fragment containing *GAP* promoter
Sig-F	ATGAGATTTCCTTCAATTTTTACTGC	Amplifying a DNA fragment containg protein secretion signal, multiple cloning site and transcription termination from plasmid pPIC9.
3AOX-TT	GATCATTAATTAACGATAAGCTTGCACAAACGAAC	Amplifying a DNA fragment containg protein secretion signal, multiple cloning site and transcription termination

		from plasmid pPIC9.
lacB-F	AAGCTATACGTATCCATCAAGCATCGTCTCAATG	Amplifying *lacB* gene
lacB-R	ATTTGCGGCCGCTTAGTATGCTCCCTTCCGCTG	Amplifying *lacB* gene
gfp-F	AAGCTATACGTAATGGTGAGCAAGGGCGAGGAG	Amplifying *gfp* gene
gfp-R	ATTTGCGGCCGCTTACTTGTACAGCTCGTCCATG	Amplifying *gfp* gene
$P_{LRA3\ (256)}$-F	GTAACGGCGCGCCAAACTGAAAGAACAAAGAACCAGTG	Amplifying a DNA fragment of putative *LRA3* promoter containing 256 bp
$P_{LRA3\ (210)}$-F	GTAACGGCGCGCCAACTGACAGAATGACTGACTCCCTA	Amplifying a DNA fragment of putative *LRA3* promoter containing 210 bp
$P_{LRA3\ (140)}$-F	GTAACGGCGCGCCGAGAAACCTCCATACGGATGATAGT	Amplifying a DNA fragment of putative *LRA3* promoter containing 140 bp
$P_{LRA3\ (120)}$-F	GTAACGGCGCGCCATAGTTAGCCCGTTCCCACCACT	Amplifying a DNA fragment of putative *LRA3* promoter

		containing 120 bp
$P_{\mathit{LRA3}\ (100)}$-F	GTAACGGCGCGCCACTTCGGCTGATTGTTAGGTTACG	Amplifying a DNA fragment of putative *LRA3* promoter containing 100 bp
$P_{\mathit{LRA3}\ (85)}$-F	GTAACGGCGCGCCTAGGTTACGTGGGGTTGAAAGAT	Amplifying a DNA fragment of putative *LRA3* promoter containing 85 bp
$P_{\mathit{LRA3}\ (48)}$-F	GTAACGGCGCGCCCTGATCCAAAGAAGGCCATTTAC	Amplifying a DNA fragment of putative *LRA3* promoter containing 48 bp
